# Efficacy of an internet-based, therapist-guided cognitive behavioral therapy intervention for adolescents and young adults with body dysmorphic disorder: a randomized controlled trial

**DOI:** 10.1186/s12888-025-06797-1

**Published:** 2025-04-14

**Authors:** Michaela Schmidt, Katrin Schoenenberg, Julia E. Engelkamp, Thomas Staufenbiel, Alexandra Martin, David D. Ebert, Andrea S. Hartmann

**Affiliations:** 1 Institute of Psychology, Department of Clinical Psychology and Psychotherapy, Osnabrück University, Osnabrück, Germany; 2 Institute of Psychology, Department of Clinical Psychology and Psychotherapy, Kiel University, Kiel, Germany; 3 Department of Psychology, Clinical Psychology and Psychotherapy of Childhood and Adolescence, Konstanz University, Universitätsstraße 10, Postbox 905, 78464 Konstanz, Germany; 4 Institute of Psychology, Department of Research Methods, Diagnostics and Evaluation, Osnabrück University, Osnabrück, Germany; 5 Department of Psychology, Clinical Psychology and Psychotherapy, University of Wuppertal, Wuppertal, Germany; 6 Department of Sport and Health Sciences, Psychology and Digital Mental Health Care, Technical University of Munich, Munich, Germany

**Keywords:** Online therapy, Cognitive behavioral therapy, Body dysmorphic disorder, Adolescents, Randomized controlled trial

## Abstract

**Background:**

Body dysmorphic disorder (BDD) is particularly prevalent yet highly understudied and undertreated in adolescence. This study evaluates the efficacy of an internet-based, therapist-guided cognitive behavioral therapy (CBT) for adolescents and young adults with BDD compared to supportive online therapy as an active control condition.

**Methods:**

In a single-blind, randomized controlled trial, *N* = 45 adolescents (aged 15–21 years) of all genders from German-speaking countries were assigned to 12 sessions of internet-based CBT (iCBT) or 12 weeks of supportive online therapy. The primary outcome was change in expert-rated BDD symptom severity from pre- to post-intervention (Yale-Brown Obsessive-Compulsive Scale Modified for Body Dysmorphic Disorder, BDD-YBOCS). Secondary outcomes included the remission and responder rate, changes in delusionality of appearance beliefs (BABS), self-rated BDD symptom severity (FKS), BDD cognitions (FKDK), quality of life (KINDL-R), and depressive symptoms (PHQ-9) from pre to post and to a 4-week follow-up.

**Results:**

iCBT was more efficient than supportive online therapy on the BDD-YBOCS (*p* =.002), with a large between-group effect size at post-intervention (Hedges’ g (*SE*) = 0.93 (0.42)), and on all secondary measures (*p* <.05), except for depressive symptoms (*p* =.068). All secondary outcome measures also showed significant improvements from pre to post iCBT, with moderate to large effect sizes, and gains were stable until the 4-week follow-up period. iCBT participants showed higher remission (61.5%) and responder rates (66.7%), compared to controls (0% and 26.7%), but only the difference in remission reached significance.

**Conclusion:**

The results indicate the efficacy of internet-based CBT in comparison to an active control condition, thus contributing to the limited intervention research in adolescent BDD and adding a much-needed treatment option. **Trial registration**: The trial was pre-registered on 2020/06/08 at the German Clinical Trials Register, DRKS00022055.

**Supplementary Information:**

The online version contains supplementary material available at 10.1186/s12888-025-06797-1.

## Background

Body dysmorphic disorder (BDD) is particularly common in adolescence, with an estimated point prevalence of 3.6% among 15-21-year-olds in a non-clinical sample in Germany [1] and 1.9% in an adolescent sample in England [2]. Moreover, this age group seems to show especially pronounced core features of BDD, such as delusionality of appearance beliefs, as well as suicidal ideation and high levels of impairment [3, 1, 4]. The first symptoms of BDD typically develop in early childhood [5, 6], with a first peak of onset at the age of 16 years [7]. Importantly, BDD seems to be chronic and unremitting if left untreated [8], with an earlier age of onset indicating an overall more severe course of the disorder [5]. The majority of young patients also meet the diagnostic criteria for at least one comorbid disorder, mainly major depressive disorder or dysthymia [6]. Given the serious long-term consequences that BDD can exert on young people’s future development, effective treatments for adolescents are urgently needed.

While cognitive behavioral therapy (CBT) and selective serotonin reuptake inhibitors (SSRIs) are considered the gold-standard treatments for adult BDD (for a meta-analytic review see Harrison, 2016; Williams et al., [9], far fewer treatment studies have focused on adolescent BDD. In the only randomized controlled trial (RCT) of CBT for adolescents with BDD conducted to date, an age-adapted CBT protocol was compared to an active control condition consisting of psychoeducational materials and weekly telephone monitoring [10]. The study involved *N* = 30 adolescents aged 12–18 and their families. The CBT protocol led to a significant improvement in BDD (pre-post within-group effect: *d* = 1.47) and related symptoms, and was found to be superior to the active control condition both at post-treatment (between-group effect: *d* = 1.13) and at 2-month follow-up (between-group effect: *d* = 0.85). At the end of treatment, 40% of adolescents in the CBT group could be classified as responders, compared to 6.7% in the active control group, indicating a clear superiority of CBT. Moreover, CBT was found to result in significant symptom improvements in an uncontrolled pilot study [11] and in several case studies [3, 12, 13] with adolescent patients. In the most recent and largest naturalistic effectiveness study in adolescents with BDD (*N* = 96), CBT was provided in two outpatient specialty clinics in Sweden and England [14]. At post-treatment, 79% of participants aged 10 to 18 years were classified as responders and 59% as full or partial remitters, and BDD symptoms continued to improve throughout the 1-year follow-up.

Although it can be concluded that effective CBT protocols for adolescent BDD do exist, only a small proportion of patients actually receive adequate help [15, 16, 4]. This is attributable to several treatment barriers, with shame, fear of stigma, and fear of discrimination being the most commonly reported [15, 16, 17]. Furthermore, individuals with BDD are often unaware or highly skeptical of the psychological nature of their concerns [15, 16, 6] and prefer to seek non-psychiatric treatments such as cosmetic and medical treatments [6, 18]. Moreover, as awareness of BDD is still low - even among medical professionals - BDD symptoms are often misdiagnosed [18] or may be mistaken for age-typical concerns. Finally, more general logistical and financial barriers can prevent individuals from seeking and receiving help, including concerns about health insurance coverage, a lack of knowledge about appropriately trained treatment providers [15, 16, 17], and long waiting times for psychotherapy [19]. Accordingly, a study by Schulte et al. [17] found that only 15.2% of German individuals with BDD were correctly diagnosed, and rates of CBT (8.4%) and psychopharmacological treatment (10%) were low. Given that younger people with BDD appear to perceive more treatment barriers, rely more on cosmetic or medical treatments, and are less likely to seek psychological treatment [17], it may be even more crucial to address these barriers in adolescents.

Internet-based CBT (iCBT) may represent a suitable way to address these needs, as it has been shown in a recent meta-analysis to result in comparable effect sizes as face-to-face CBT [20], with therapist guided interventions showing superior results to automatic guided interventions [21]. At the same time iCBT reduces prominent BDD-specific treatment barriers associated with face-to-face contact [22].

Several studies with adult populations have demonstrated the applicability and efficacy of iCBT for BDD. For instance, Enander et al. [23–25] tested a therapist-guided iCBT called *BDD-NET*, which is available in both Swedish and English and is currently the best evaluated iCBT program for BDD. The intervention yielded significant improvements in BDD symptom severity from baseline to 6-month follow up (*d* = 1.42) and was found to be superior to an active control condition (supportive online therapy; *d* = 0.87 at 6-month follow up). Using a different approach, Wilhelm et al. [26] developed the first smartphone-based application for adults with BDD and confirmed its superiority over a waitlist control group in an RCT. In Germany, Schoenenberg et al. [27] tested the feasibility and efficacy of an internet-based treatment for adults with clinical and subclinical BDD (*imagin*). Although adherence to the intervention was low and the dropout rate high (55.6%), the program content and usability were rated highly, and participants’ satisfaction with their appearance improved significantly.

For adolescent BDD, only one iCBT [28] has been evaluated in terms of its feasibility and long-term efficacy with *N* = 20 participants ages 12–17 and their caregivers. The program was found to be both credible and satisfactory, and was associated with a strong and statistically significant reduction in BDD symptom severity from pre- to post-therapy (*d* = 2.29), even after a 12-month follow-up (*d* = 2.94). At the primary endpoint (3-month follow-up) 73.7% of participants could be classified as responders and 63.2% as remitters. Moreover, several meta-analyses of iCBT for adolescents with other mental disorders, which show some symptom overlap with BDD, have confirmed the efficacy of internet-based programs in reducing anxiety, depression [29, 30], eating disorders, and obsessive-compulsive disorder [31], suggesting its potential as a low-threshold alternative to face-to-face therapy. However, the comparative effectiveness of iCBT versus a control condition in adolescents with BDD has not yet been established.

In view of the paucity of intervention research into adolescent BDD and the urgent need for adequate, low-threshold treatment options for this age group, we developed a therapist-guided iCBT intervention for adolescents and young adults with BDD (*ImaginYouth*) and evaluated its efficacy in comparison to an active control condition (supportive online therapy) in a single-blind two-arm RCT. Specifically, we hypothesized that the iCBT intervention would lead to a significant reduction in expert-rated BDD symptom severity from pre- to post-intervention as the primary outcome. Furthermore, we expected the BDD symptom severity at post-intervention to be significantly lower in the iCBT condition than in the active control condition. In terms of secondary outcomes, we hypothesized that the iCBT intervention would lead to a significant reduction in self-rated BDD symptom severity, delusionality of appearance beliefs, and comorbid symptoms of depression and to a significant improvement in patients’ quality of life. Moreover, we expected that severity of BDD, BDD cognitions, delusionality, and depression would be significantly lower and quality of life would be significantly higher in the iCBT condition than in the active control condition at post-intervention, and that these differences would remain stable over a 4-week follow-up period. Finally, we assumed that the remission and responder rates at post-treatment would be significantly greater in the iCBT condition than in the active control condition.

## Method

The trial was pre-registered at the German Clinical Trials Register (DRKS00022055) prior to data collection on 2020/06/08. The study protocol has been previously published [32]. The trial is described in accordance with the CONSORT 2010 guidelines for reporting parallel group randomized trials [33].

### Participants

German-speaking participants of any gender, aged between 15 and 21 years with a primary diagnosis of BDD according to the Diagnostic and Statistical Manual of Mental Disorders, 5th edition (DSM-5) were eligible to participate. Comorbid diagnoses were permitted. The exclusion criteria were current substance abuse, bipolar or psychotic episodes, suicidal intentions and plans, current outpatient or inpatient psychotherapy, a change in psychopharmacological medication in the past two months, and/or a limitation of cognitive abilities that would interfere with work on the intervention.

### Recruitment

Recruitment took place from 07/2020 to 09/2022 in German-speaking countries (i.e., Germany, Austria, Switzerland) via social media platforms (e.g., Instagram, Facebook, YouTube, Twitter/X, TikTok), the study’s website, university email distribution lists, posters, flyers, and press releases. Additionally, outpatient child and adolescent psychotherapists as well as student counselling centers were provided with information about the study and encouraged to refer waiting-list patients to our program. Participants were self-selected into the study and were not compensated for their participation.

### Study design and procedure

This single-blind, two-arm RCT was conducted from 07/2020 (start of recruitment) to 09/2023 (last follow-up assessment after reaching the calculated sample size according to the a-priori power analysis). After initial contact by telephone, email, or our social media platforms, participants completed a brief online self-report screening (via Unipark; EFS Survey, Questback GmbH, Cologne) to assess basic inclusion criteria (e.g., age, no current psychotherapy). If these requirements were met, participants were contacted by one of the study’s diagnosticians (a clinical psychologist in training, supervised and trained by the first and last author) to schedule an online appointment for the informed consent procedure and diagnostic interviews. The diagnostician assessed the primary diagnosis of BDD, the BDD symptom severity, delusionality of appearance beliefs, comorbid disorders, as well as full study eligibility. All interviews were conducted using the secure video consultation tool RedConnect (RED Medical Systems GmbH, Munich). Participants were given comprehensive verbal and written information about the study procedure, our privacy policy, and the possibility to withdraw from the intervention at any time without consequence. Prior to the diagnostic interviews, they were required to sign an informed consent document and to return it to the diagnostician by email. We did not obtain consent from the participants’ legal guardians, as adolescents over the age of 15 can participate in psychotherapy in Germany without parental consent (§ 36 SGB 1). Diagnostic sessions were recorded and interrater reliability (ICC (3, 2); Shrout & Fleiss [34], was calculated in 20% of randomly selected cases.

If all inclusion criteria were met, participants were randomly assigned to one of two intervention conditions by our external collaborators (second author). Full randomization (no stratification or blocking) of the prospective 40 cases was generated by the second author using a list randomizer (RANDOM.ORG, Randomness and Integrity Services Ltd, Dublin). The diagnostician was blinded to the randomization process at pre- and post-intervention. However, both the participants and the study therapist (first author), who enrolled participants to the condition, were aware of condition assignment, as has been the practice in previous RCTs of BDD CBTs [23, 10, 26]. If participants switched over to the iCBT condition after completing the active control condition and all subsequent assessment points, it was no longer possible to maintain diagnostician blinding during their following pre- and post-iCBT diagnostic interviews.

After randomization, all participants completed an online questionnaire battery and were then provided with a login to the e-mental health platform Minddistrict (Minddistrict, Berlin), where they were able to access the intervention to which they had been assigned. Participants in the iCBT condition completed a short online questionnaire battery after each of the 12 sessions (i.e., 12 questionnaires in total) and a more comprehensive online questionnaire battery after 6 sessions, at post-intervention and at a 4-week follow-up. Post measures were only administered following completion of all 12 sessions. At this point, another structured interview was conducted by the blinded diagnostician. Participants in the active control condition completed the same 12 questionnaires each week for a total of 12 weeks, as well as the more comprehensive questionnaire battery after 6 weeks, at post-intervention and at the 4-week follow-up, resulting in the same set of questionnaires being administered in both conditions. Equivalent to the iCBT condition, participants in the active control condition participated in the structured clinical interview at post-intervention. After the 4-week follow-up and all corresponding measures, participants in the active control condition were able to cross over to the iCBT condition. The follow-up was set at 4 weeks, as this was considered the maximum ethical length of time for active control participants to wait before switching to the iCBT condition.

### Interventions

A detailed overview of the development and content of the interventions can be found in the study protocol [32].

#### ImaginYouth

The layout and some core elements of the iCBT intervention were inspired by a therapist-guided iCBT for adults with clinical and subthreshold BDD [27], with substantial modification and adaptation for youth and young adults. The final intervention consisted of 12 interactive self-help sessions and encompassed six core modules of CBT for BDD in the following order: (1) psychoeducation; (2) cognitive restructuring; (3) self-deprecating thoughts and self-esteem; (4) safety behaviors and exposure and response prevention; (5) mirror retraining; (6) relapse prevention. Each session was followed by corresponding homework assignments to apply the session contents in daily life, as well as written feedback on the session assignments and homework from the study therapist (first author, who is a clinical psychologist in training, supervised by the last author in biweekly supervision sessions). The study therapist also checked for compliance with the session and assignments. Only after a session had been completed, including homework, could participants begin the next session. Participants were encouraged to complete one session per week, resulting in an ideal total intervention duration of 12 weeks. However, they were not excluded from the analyses if they took longer to complete the intervention. Between sessions, participants could communicate freely with the study therapist via an integrated chat function at any time. Session assignments, homework and messages from the participants needed to be reviewed and responded to by the study therapist within 36 h, except on weekends. None of the participants had any face to face contact with the study therapist. Both the contents of the sessions, including all additional worksheets, and communication with the study therapist were conducted in German.

#### Supportive online therapy

A supportive online therapy according to the definition of the American Psychological Association (2018) was selected as the active control condition. Supportive online therapy had been shown to be feasible as a control condition in previous RCTs of iCBT for adults with BDD [23] and for children and adolescents with BDD, depression or anxiety symptoms [35, 10, 36]. It served as a control for therapist attention and the possible anxiety-reducing effect of sharing distress with the therapist [23]. Participants were aware of treatment assignment, but not that the supportive online therapy was merely an active control for the ImaginYouth condition to reduce expectancy effects. To date, there are no reports of the efficacy of supportive online therapy in the treatment of body dysmorphic disorder.

The active control condition included written psychoeducational information about BDD, equivalent to the psychoeducational material of the first module of the ImaginYouth intervention (BDD symptoms, prevalence, development, maintenance and treatment options). However, as opposed to the ImaginYouth condition, there were no exercises to facilitate the transfer to the patient’s individual case or to identify the intervention targets. The reading material was divided into 6 chapters and was freely accessible for 12 weeks. In addition, participants were contacted weekly by the study therapist via the program’s messaging function and encouraged to talk freely about their current well-being as well as their experiences, thoughts, and feelings about BDD symptoms and how these affect their lives. Therapeutic techniques were limited to low-threshold counseling (e.g., validation, empathy, encouraging self-care and mood stabilization). Participants’ messages had to be checked and responded to within 36 h. Supportive online therapy was provided by the same therapist who delivered the ImaginYouth intervention. To ensure treatment integrity, topics discussed during the supportive communication between the study therapist and the participants were monitored by the last author in biweekly supervision sessions with the study therapist. The language of communication and the psychoeducational material was German. Again, the participants had no face to face contact with the study therapist.

### Safety management

We obtained contact information (full name, address, telephone number, email) from all participants before the intervention began to enable us to reach them in the event of an emergency. Individuals who indicated specific suicidal intentions and plans in the online screening or diagnostic interviews were excluded from participation. During the intervention, suicidality was monitored after each session in the iCBT condition [37] or weekly in the active control condition, using a brief online questionnaire (PHQ-9; Kroenke et al. [38] suicide item of the BDI-2; Hautzinger et al., [37]. In the event of acute suicidal intentions, a stepped emergency plan was initiated by the study therapist under the guidance of the study supervisor. Participants who were unable to distance themselves from suicidal intentions were accompanied until they were safe (e.g. in the care of the police, the emergency services or in a psychiatric clinic) and then excluded from the study. A detailed overview of our safety and quality management can be found in the study protocol [32].

### Measures

A detailed overview of all outcome measures used in this study can be found in the study protocol [32]. The measures analyzed in this manuscript are described below.

#### Primary outcome measure

**Yale-Brown Obsessive-Compulsive Scale Modified for Body Dysmorphic Disorder (BDD-YBOCS)**. The BDD-YBOCS (German-language version: Stangier et al., [39] is the gold-standard semi-structured expert interview to evaluate BDD symptom severity. It was also used in the present study to assess the responder rate (defined as a 30% reduction in BDD-YBOCS score from pre- to post-intervention; Fernández de la Cruz, 2021) and remission status (defined as a BDD-YBOCS score of ≤ 16 at post-intervention; Fernández de la Cruz, 2021). Total scores range from 0 to 48, with higher scores indicating more severe BDD. The scale has shown good internal consistency in an adolescent sample (α = 0.87–0.96; Monzani et al., [40] current study: α = 0.81), and the interrater reliability in the current study was excellent (ICC (3, 2) = 0.92; 95% CI: 0.69, 0.98).

#### Secondary outcome measures

**Brown Assessment of Beliefs Scale (BABS)**. The BABS (German-language version: Buhlmann [41], is an expert-rated clinical interview that measures the delusionality of appearance beliefs. Total scores range from 0 to 24, with higher scores indicating more delusional thinking. Internal consistency in the German validation study with an adult sample was good (α = 0.89; Buhlmann [41], current study: α = 0.84), and the interrater reliability in the current study was excellent (ICC (3, 2) = 0.97; 95% CI: 0.92, 0.99).

**Body Dysmorphic Symptoms Inventory ([Fragebogen Körperdysmorpher Störung]**,** FKS)**. The FKS [42] measures the severity of BDD symptoms, with higher total scores indicating more severe BDD symptoms (range: 0 to 64). The scale showed good internal consistency in a German adult sample (*α* = 0.88; Buhlmann & Wilhelm [42], current study: α = 0.68).

**Questionnaire of body-dysmorphic cognitions ([Fragebogen Körperdysmorpher Kognitionen]**,** FKDK)**. The FKDK [43] assesses the frequency and conviction of BDD-related cognitions. A mean value is calculated for each subscale, with higher scores indicating more frequent (range: 1 to 5) or more intense (range: 1 to 100) BDD cognitions. The two subscales showed very good internal consistency in a German adult sample ($\:\omega\:$ = 0.90 for the frequency subscale and $\:\omega\:$ = 0.96 for the conviction subscale; Schüller et al., [43] current study: frequency subscale $\:\omega\:$ = 0.87, conviction subscale

$$\:\omega\:$$ = 0.93).

**Generic quality of life instrument for children and adolescents– revised ([Fragebogen zur Lebensqualität von Kindern und Jugendlichen**,** revidierte Fassung]**,** KINDL-R)**. The KINDL-R [44] measures child and adolescent quality of life. Total scores range from 0 to 120, with higher scores indicating better quality of life. The total scale has shown good internal consistency in a German pediatric sample (α = 0.95; Ravens-Sieberer & Bullinger [44], current study: α = 0.84).

**Patient Health Questionnaire (PHQ-9)**. The PHQ-9 [38] assesses the severity of depression with nine items corresponding to the depression criteria of the DSM-IV. A higher total score indicates higher symptom severity (range: 0 to 27). Internal consistency was good in the original validation with an adult sample (α = 0.89; Kroenke et al., [38] current study: α = 0.82).

**Sociodemographic and clinical characteristics**. Self-report data were gathered on age, gender, nationality, ethnicity, comorbidity, duration of illness, and current drug treatment. Due to an error, two questions regarding nationality and ethnicity were not included in the original demographic questionnaire but were sent to participants after the study began, resulting in a lower number of completed responses for these questions.

### Statistical analyses

All analyses were performed using SPSS Statistics (Version 26; IBM; Armonk, New York, USA). We performed a linear mixed models analysis, which included all randomized participants regardless of whether they completed the intervention, and a per protocol analysis which only included participants who completed the intervention as well as the pre, post, and 4-week follow-up questionnaires and the pre and post diagnostic interviews.

For the former, linear mixed models (LMM) with random intercepts for subjects were used to account for interindividual differences. LMM have several advantages over traditional mixed ANOVA methods for analyzing longitudinal data [45]. One advantage is that LMM can deal with missing data without excluding entire cases from the analysis. All available data are used directly to estimate model parameters, taking into account the nested structure of the data (time nested within subjects) through multilevel modeling. As a result, the power of LMM is higher compared to mixed ANOVA [46]. All LMM models were implemented using the restricted maximum likelihood (REML) method. The LMM analyses included all main and interaction effects as fixed effects. Missing data was not imputed before applying LMM, as this reduces power [47].

As the effect size Cohen’s *d* is biased especially for smaller samples, the effect size Hedges’ *g* is reported instead. The differences between the means are first normalized by a standard deviation and then corrected according to Hedges [48], formula (6e)). The pooled variances of both groups were used as standard deviations for between-group comparisons and as standard deviations of the differences for within-group comparisons. In addition, standard errors are given for all effect size measures [49], formulas (5), (6a&b) and (8b)).

To prevent further confounding influences of the missing data on the effect sizes, we used multiple imputation to calculate the effect sizes. More specifically, we generated 20 imputed data sets using the predictive mean matching method [50], then calculated the effect size measures for all 20 completed data sets and reported them aggregated using Rubin’s rules [51]. All dependent variables examined, group membership and the socio-demographic variables age and gender were used to estimate the imputations.

For the per-protocol analysis, we analyzed the efficacy of the iCBT intervention and its comparison to the active control condition by performing a 2 (group) x 3 (time) analysis of variance (ANOVA) with Huynh-Feldt correction for each self-rated independent variable (FKS, FKDK, KINDL-R, PHQ-9). For the diagnostic interviews conducted at pre- and post-intervention, a 2 (group) x 2 (time) ANOVA was performed. Pairwise simple contrasts were computed for both the between-group difference at post-treatment and follow-up, and the within-group difference from pre- to post-intervention and from pre- to follow-up. Effect sizes were reported as partial eta squared (_p_η^2^) for mixed ANOVAs and Hedges’ g for between- and within-group differences.

Fisher’s exact tests or t-tests for independent groups were used to compare groups with respect to remission and responder status, dropout rate, and clinical and demographic characteristics at baseline.

### Sample size calculation

The a priori power analysis (G*Power; Faul et al., [52] focused on the number of participants required to test whether ImaginYouth was more effective than the active control condition in terms of reducing BDD symptom severity on the BDD-YBOCS, i.e., the interaction effect in the 2 × 2 ANOVA. The power analysis was based on *α* = 0.05 and 1-*β* = 0.95, and an effect size of *f* = 0.36, derived from a meta-analysis on effects in internet-based intervention studies with adolescents [30]. This resulted in a total required sample size of *n* = 28, i.e. *n* = 14 per study arm. As internet-based studies are expected to have a dropout rate of approximately 30% [53], we aimed to recruit 20 patients per study arm.

Owing to high attrition within the iCBT condition and challenges in recruiting participants, randomization was halted once a sample size of 16 participants was reached in the active control group. Subsequently, all interested participants (*n* = 17) were directly assigned to the iCBT condition. The blinded diagnostician was not made aware of this change, thereby preserving the integrity of the blinding. There was also no information about this to the participants.

## Results

### Baseline demographic and clinical characteristics

The randomized participants age ranged from 16 to 21, with a median age of 20 years (*M* = 19.38). They were mainly female (80%), German (100%) and not part of an ethnic minority (4.3%). On average, participants had already suffered from BDD for 6.45 years before starting the trial, with an average age of onset of 13 years (median: 14 years). Participants had poor insight (BABS mean score: 14.47) and at least one comorbid disorder (68.9%), with depression (30.95%), social phobia (28.57%) and unspecified eating disorder (9.52%) being the most common ones. BDD-YBOCS total scores ranged from 15 to 44, with a mean score of 28.95 (median: 28.50). 16% of participants were on a psychopharmaceutical treatment when entering the trial. Detailed demographic and clinical characteristics of the randomized sample und the individual condition samples are provided in Table 1.

**Table 1:** Baseline demographic and clinical characteristics of randomized participants

Variables	Randomized (*N* = 45)	Imagin Youth (*n* = 29)	Active Control (*n* = 16)
	*M* (*SD*)	*M (SD)*	*M (SD)*
Age (years)	19.38 (1.70)	19.24 (1.79)	19.56 (1.46)
BDD YBOCS (sum score)	28.95 (6.29)	28.61 (6.53)	29.56 (6.00)
BABS (sum score)	14.43 (5.11)	14.82 (5.43)	13.75 (4.56)
FKDK conviction (mean score)	45.99 (20.49)	43.20 (18.73)	51.37 (23.24)
FKDK frequency (mean score)	3.08 (0.65)	3.09 (0.52)	3.06 (0.87)
FKS (sum score)	37.42 (6.08)	38.07 (6.11)	36.25 (6.06)
PHQ-9 (sum score)	11.89 (5.27)	11.90 (5.49)	11.87 (5.01)
KINDL-R (sum score)	46.00 (11.22)	45.70 (12.02.)	46.53 (9.98)
Number of comorbid disorders	1.07 (1.00)	1.0 (1.12)	1.19 (0.83)
Duration of BDD (years)	6.45 (3.24)	6.79 (3.46)	5.88 (2.83)
	*n (%)*	*n (%)*	*n (%)*
Gender (female)	36 (80)	23 (79.3)	13 (81.3)
Nationality (German)^†^	23/23 (100)	13/13 (100)	10/10 (100)
Part of an ethnic minority (yes)^†^	1/23 (4.35)	0/13 (0)	1/10 (10)
Current pharmacologic treatment	7 (15.90)	5 (17.9)	2 (12.5)
Comorbidity (current)			
At least 1 comorbid disorder	30 (68.18)	17 (60.7)	13 (81.3)
Depression	13 (29.55)	9 (32.14)	4 (25)
Social Phobia	12 (27.27)	5 (17.85)	7 (58.3)
Bulimia Nervosa	2 (4.54)	0 (0)	2 (12.5)
Eating disorder, unspecified	4 (9.09)	4 (14.29)	0 (0)
PTSD	1 (2.27)	1 (3.57)	0 (0)
Hypersomnia	1 (2.27)	0 (0)	1 (8.3)
Insomnia	1 (2.27)	0 (0)	1 (8.3)
Panic Disorder	2 (4.54)	2 (7.14)	0 (0)
OCD	2 (4.54)	2 (7.14)	0 (0)
ADD	1 (2.27)	1 (3.57)	0 (0)
Exam Anxiety	2 (4.54)	1 (3.57)	1 (8.3)
Insight			
Good	16 (36.36)	10 (35.7)	6 (37.5)
Poor	13 (29.55)	7 (25.0)	6 (37.5)
No insight	15 (34.10)	11 (39.3)	4 (25)

Note. BDD, body dysmorphic disorder; BDD-YBOCS, Yale-Brown Obsessive-Compulsive Scale Modified for Body Dysmorphic Disorder; BABS, Brown Assessment of Beliefs Scale; FKS, Body Dysmorphic Symptoms Inventory; FKDK, Questionnaire of body-dysmorphic cognitions; PHQ-9, Patient Health Questionnaire; KINDL-R, Generic quality of life instrument for children and adolescents– revised; *M*, mean; *SD*, standard deviation; PTSD, Post-traumatic stress disorder; OCD, Obsessive-compulsive disorder; ADD, Attention deficit disorder.

^†^ Lower number of completed responses due to an error in the original demographic questionnaire

### Attrition and adherence to the study protocol

**Fig. 1:**
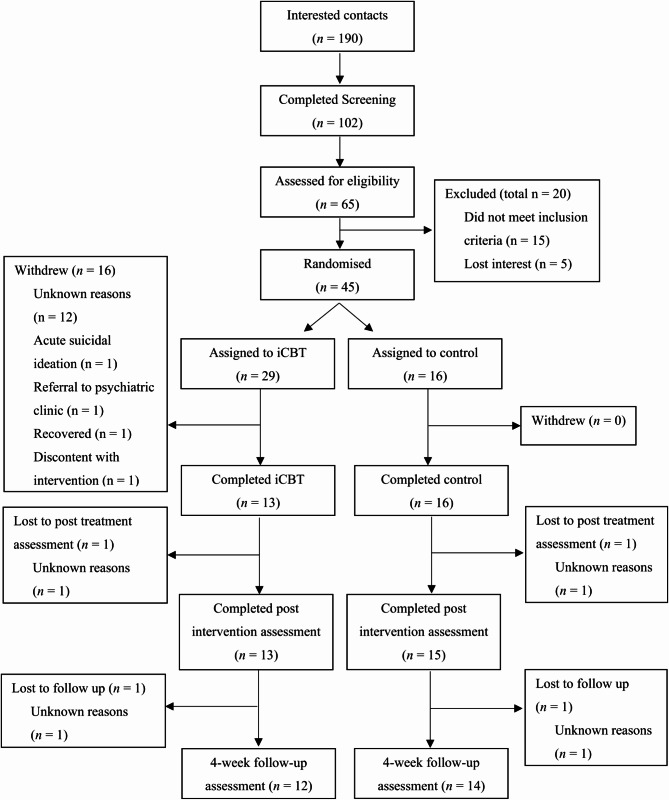
Flow of participants through the trial. *Note*: iCBT: internet-based cognitive behavioral therapy; control: active control group

Figure 1 shows the flow of participants through the trial. In the iCBT condition, 13 out of 29 randomized participants completed the intervention and as many completed the post-assessment (44.8%). In the active control condition, all 16 participants who started the supportive online intervention completed it, and 15 completed the post-assessment (93.8%), resulting in a significantly higher attrition rate in the iCBT condition than in the active control condition (*p* =.001, φ = 0.48). At the 4-week follow-up, 12 out of 29 (41.37%) participants in the iCBT condition and 14 out of 16 (87.5%) participants in the active control condition had completed measures at all three time points (pre, post, follow-up).

As monitored by the study therapist, both interventions were executed as intended. Notably, participants in the iCBT condition did not complete the intervention within 12 weeks (84 days; 1 session per week) as recommended, but took a mean of *M* = 180.69 days.

(*SD* = 53.11; median = 189 days; range = 116–267 days). Study therapists’ adherence to the study protocol was monitored and confirmed by the study supervisor (last author, clinical psychologist) during biweekly supervision sessions.

### Remission and responder rate

Based on the definition provided by Fernández de la Cruz (2021), the responder rate was defined as a reduction of 30% or more in the score of our primary measure outcome (BDD-YBOCS) from pre- to post-intervention, while remission status was characterized by a score of 16 or lower on the same measure at post-intervention. The two groups differed significantly regarding the remission rate (*p* >.001, φ = 0.68) but not regarding the responder rate (*p* =.057, φ = 0.40). In the iCBT condition, 66.7% of participants reached responder status and 61.5% reached remission status. In the active control condition, 26.7% reached responder status and 0.0% reached remission status.

### Adverse effects

One adverse effect, i.e., an incident that participants directly attributed to their participation in the study intervention, such as sleep disturbances, increased stress levels, or relationship problems (Aronson, 2023), was reported during the iCBT intervention. Possible approaches to address this were discussed with the participant via chat by the study therapist. Furthermore, two exacerbations of suicidality happened over the course of the intervention in two cases, without the participants directly linking them to their participation in the study. In response, the stepped emergency plan was successfully implemented: one participant ceased reporting suicidal ideation, while the other was taken to a psychiatric clinic by their mother.

### LMM analysis

#### Primary outcome

For expert-rated BDD symptom severity, a significant time x group interaction effect favoring the iCBT condition was observed (BDD-YBOCS: *F*(1, 29.87) = 11.47, *p* =.002). While BDD-YBOCS scores decreased significantly from pre- to post-intervention in both conditions (iCBT: *p* <.001, Hedges’ g (*SE*) = 1.24 (0.56); active control: *p* =.018, Hedges’ g (*SE*) = 0.73 (0.23)), the scores were significantly lower in the iCBT condition than in the active control condition at post-intervention (*p* <.001, Hedges’ g (*SE*) = 0.93 (0.42)). For a detailed overview of estimated means, standard errors and pairwise within- and between-group effect sizes, see Table 2.

**Table 2:** Results of the intention-to-treat analysis (all randomized cases): Changes in outcome measures

				Within-group differences				Between-group differences			
	Pre-treatment	Post-treatment	Follow-up	Pre-treatment to post		Pre-treatment to follow-up		Post-treatment		Follow-up	
	EMM (*SE*)	EMM (*SE*)	EMM (*SE*)	*M*diff (95% CI)	Hedges’ g (*SE*)	*M*diff (95% CI)	Hedges’ g (*SE*)	*M*diff (95% CI)	Hedges’ g (*SE*)	*M*diff (95% CI)	Hedges’ g (*SE*)
BDD-YBOCS											
iCBT	28.70 (1.32)	16.34 (1.77)	-	-12.36 (-15.88; -8.85)***	1.24 (0.56)	-	-	9.00 (3.97; 14.03)***	0.93 (0.42)	-	-
Active control	29.56 (1.75)	25.34 (1.79)	-	-4.22 (-7.65; -0.79)*	0.73 (0.23)	-	-	-	-	-	-
BABS											
iCBT	14.74 (0.94)	11.12 (1.17)	-	-3.62 (-5.70; -1.54)**	0.48 (0.31)	-	-	2.61 (-0.84; 6.06)	0.37 (0.40)	-	-
Active control	13.75 (1.25)	13.73 (1.27)	-	-0.23 (-2.00; 1.95)	0.03 (0.18)	-	-	-	-	-	-
FKS											
iCBT	38.07 (1.47)	21.90 (1.93)	20.24 (1.98)	-16.17 (-19.68; -12.65)***	1.50 (0.62)	-17.83 (-21.45; -14.19)***	1.51 (0.69)	8.48 (2.96; 13.99)**	0.75 (0.38)	10.08 (4.40;15.75)***	0.73 (0.43)
Active control	36.25 (1.98)	30.37 (1.98)	30.32 (2.05)	-5.88 (-9.22; -2.53)***	1.01 (0.26)	-5.93 (-9.44; -2.42)**	0.80 (0.25)	-	-	-	-
FKDK conviction											
iCBT	43.20 (3.82)	25.77 (4.75)	28.08 (4.85)	-17.43 (-25.26; -9.59)**	0.56 (0.33)	-15.12 (-23.20; -7.04)**	0.48 (0.37)	21.43 (7.47; 35.39)**	0.83 (0.39)	13.51 (-0.77; 27.79)	0.44 (0.39)
Active control	49.46 (5.20)	47.20 (5.14)	41.59 (5.27)	-2.23 (-9.77; 5.26)	0.19 (0.16)	-7.86 (-15.75; 0.03)	0.40 (0.19)	-	-	-	-
FKDK frequency											
iCBT	3.09 (0.14)	2.30 (0.17)	2.27 (0.17)	-0.79 (-1.07; -0.51)***	1.03 (0.50)	-0.82 (-1.10; -0.55)***	0.75 (0.41)	0.48 (-0.02; 0.98)	0.83 (0.39)	0.41 (-0.10; 0.91)	0.44 (0.39)
Active control	3.01 (0.18)	2.78 (0.18)	2.68 (0.19)	-0.23 (-0.48; 0.03)	0.47 (0.18)	-0.33 (-0.60; -0.06)*	0.56 (0.21)	-	-	-	-
PHQ-9											
iCBT	11.90 (1.07)	7.77 (1.35)	9.36 (1.38)	-4.12 (-6.44; -1.81)***	0.42 (0.28)	-2.53 (-4.92; -0.15)*	0.23 (0.24)	3.79 (-1.44; 7.72	0.46 (0.39)	1.74 (-2.29; 5.78)	0.15 (0.37)
Active control	11.88 (1.46)	11.56 (1.44)	11.11 (1.48)	-0.31 (-2.55; 1.92)	0.08 (0.17)	-0.77 (-3.11; 1.57)	0.13 (0.21)	-	-	-	-
KINDL-R											
iCBT	45.75 (2.36)	57.31 (2.98)	54.71 (2.98)	11.56 (6.42; 16.70)***	-0.64 (0.36)	8.96 (3.82; 14.10)***	-0.41 (0.33)	8.62 (17.21; 0.04)*	-0.58 (0.42)	5.33 (-3.37; 14.03)	-0.18 (0.38)
Active control	46.36 (3.14)	48.69 (3.10)	49.38 (3.19)	2.33 (-2.35; 7.00)	-0.34 (0.19)	3.02 (-1.90; 7.93)	-0.25 (0.18)	-	-	-	-

Note. iCBT, internet-based cognitive behavioral psychology; BDD-YBOCS, Yale-Brown Obsessive-Compulsive Scale Modified for Body Dysmorphic Disorder; BABS, Brown Assessment of Beliefs Scale; FKS, Body Dysmorphic Symptoms Inventory; FKDK, Questionnaire of body-dysmorphic cognitions; PHQ-9, Patient Health Questionnaire; KINDL-R, Generic quality of life instrument for children and adolescents– revised; EMM, estimated marginal mean; *SE*, standard error; *M*diff, mean group difference.

*p <.05, **p <.01, ***p <.001

#### Secondary outcomes

We observed significant time x group interaction effects in favor of the iCBT condition for all secondary measures, i.e., delusionality of appearance beliefs (BABS: *F*(1, 27.08) = 6.63, *p* =.016), BDD symptom severity (FKS: *F*(2, 58.22) = 13.91, *p* <.001), conviction of BDD cognitions (FKDK conviction subscale: *F*(2, 53.89) = 3.92, *p* =.026), frequency of BDD cognitions (FKDK frequency subscale: *F*(2, 52.48) = 5.32, *p* =.008), and quality of life (KINDL-R: *F*(2, 52.40) = 3.66, *p* =.033), with the exception of depressive symptoms (PHQ-9: *F*(2, 53.10) = 2.83, *p* =.068). All secondary measures, including depression, improved significantly from pre- to post-iCBT (< 0.001 ≤ *p* ≤.001; Hedges’ g = 0.42–1.50) and from pre-iCBT to follow-up (< 0.001 ≤ *p* ≤.038; Hedges’ g = 0.23–1.51). A detailed overview of estimated means, standard errors and pairwise within- and between-group comparisons is provided in Additional file 1 (see: Additional file 1: Results of the per-protocol analysis: Changes in outcome measures).

### Per-protocol analysis

#### Primary outcome

Overall, the results for both primary and secondary outcomes were consistent with the ITT analysis. For expert-rated BDD symptom severity, a significant time x group interaction effect favoring the iCBT condition was observed (BDD-YBOCS: *F*(1, 25) = 10.700, *p* =.003;

_p_η^2^ = 0.300). BDD-YBOCS scores decreased significantly from pre- to post-intervention in both conditions (iCBT: *p* <.001, Hedges’ g = 1.56; active control: *p* =.018,

Hedges’ g = 0.72), but the scores were significantly lower in the iCBT condition than in the active control condition at post-intervention (*p* =.006). For a detailed overview of estimated means, standard errors and pairwise within- and between-group effect sizes, see Table S1 (Additional file 1).

#### Secondary outcomes

Similar to the ITT analysis, we observed significant time x group interaction effects in favor of the iCBT condition for all secondary measures, i.e., delusionality of appearance beliefs (BABS: *F*(1,25) = 6.02, *p* =.021; _p_η^2^ = 0.194), BDD symptom severity (FKS: *F*(2, 48) = 11.50, *p* <.001; _p_η^2^ = 0.324, ε_HF_ = 0.778), conviction of BDD cognitions (FKDK conviction subscale: *F*(2, 46) = 3.58, *p* =.036; _p_η^2^ = 0.135, ε_HF_ = 1.000), frequency of BDD cognitions (FKDK frequency subscale: *F*(2, 44) = 6.05, *p* =.006; _p_η^2^ = 0.216, ε_HF_ = 1.000), and quality of life (KINDL-R: *F*(2, 42) = 3.42, *p* =.049; _p_η^2^ = 0.140, ε_HF_ = 0.873), with the exception of depressive symptoms (PHQ-9: *F*(2,46) = 2.36, *p* =.110; _p_η^2^ = 0.093, ε_HF_ = 0.943). All secondary measures, including depression, improved significantly from pre- to post-iCBT (< 0.001 ≤ *p* ≤.017; Hedges’ g = 0.64–1.65) and from pre-iCBT to follow-up (< 0.001 ≤ *p* ≤.017; Hedges’ g = 0.87–1.82), except for depressive symptoms (*p* =.097; Hedges’ g = 0.43). A detailed overview of estimated means, standard errors and pairwise within- and between-group comparisons can be found in Table S1 (see Additional file 1).

## Discussion

This is the first study to evaluate the efficacy of iCBT for BDD and to compare it to an active control condition using a gold-standard single-blind RCT in the understudied, yet highly affected, adolescent population. As treatment rates are extremely low among individuals with BDD, the study aimed to evaluate an alternative treatment option that addresses typical treatment barriers. Overall, the iCBT intervention ImaginYouth was associated with significant and large improvements in all expert-rated and self-rated measures, including BDD symptom severity, frequency and conviction of BDD cognitions, delusionality of appearance beliefs, quality of life, and highly comorbid symptoms of depression. Within-group effect sizes on all measures were moderate to large at post-treatment (Hedges’ g = 0.42–1.50), and the improvements on all self-report measures remained stable over a 4-week follow-up period. At the end of the ImaginYouth intervention, two thirds of the participants were classified as responders and almost as many were classified as remitters. This is also reflected in the mean BDD-YBOCS score at post-treatment (*M* = 16.31, *SD* = 7.73), which was almost half of the mean score at baseline and no longer supports a diagnosis of BDD [54]. Importantly, ImaginYouth appeared to result in better outcomes than a supportive online intervention, consisting of extensive psychoeducational reading material on BDD as well as weekly supportive online contacts with a clinical psychologist, i.e. a very conservative control group. The benefits of ImaginYouth were demonstrated by all expert-rated measures (BDD symptom severity, delusionality) at post-intervention and by all self-rated measures (BDD symptom severity, frequency and conviction of BDD cognitions, quality of life) at post-intervention and at 4-week follow-up, with the exception of depressive symptoms. The latter declined significantly over the course of ImaginYouth, but improvements did not reach significant differences compared to the active control condition. Similar effects have been reported in previous iCBT trials for adult BDD using similar active control conditions [23]. Overall, these results underscore the efficacy of ImaginYouth as a BDD-specific treatment protocol.

The results found in the present study are largely consistent with or even exceed those from previous studies evaluating face-to-face CBT for adolescents and adults with BDD [11, 55, 56, 13, 14], which reported responder rates ranging from 40 to 79% and post-treatment within-group effect sizes ranging from 0.83 to 1.80. Moreover, our findings are comparable to the only other study on iCBT for adolescent BDD (responder rate: 73.7%, remission rate: 63.2%; Rautio et al., [28] and to established iCBT programs for adult BDD (responder rate: 82%; Enander et al., [24]. Furthermore, the present results exceed the meta-analytical findings regarding iCBT for adolescents with symptomatically similar disorders such as depression (pooled within-group effect size: 0.51), anxiety (pooled within-group effect size: 0.44) or eating disorders (within-group effect sizes: 0.16–0.39) [29, 30, 31]. However, these comparisons need to be made with caution due to differences in the recruited samples and treatment protocols.

It is important to note that our sample showed moderate symptom severity, which was comparable to other iCBT studies with adult and adolescent BDD patients [23, 28] and previous face-to-face CBT studies with an adult BDD sample [9], but less severe than in previous face-to-face CBT studies with adolescent BDD patients [11, 10]. Furthermore, our participants were aged between 15 and 21 years (*M* = 19.38), which is slightly older than the samples in previous treatment studies of adolescent BDD (*M* = 15.23 to *M* = 16.2 years; Greenberg et al., [11, 56, 10, 28]. In addition, our sample consisted predominantly of female participants (80.0%), which may limit the generalizability of the findings. Nevertheless, the gender ratio of our sample is consistent with similar studies on adolescent BDD [10, 28, 14], and the only study on gender-specific prevalence rates of adolescent BDD confirmed higher rates of BDD in girls than in boys [2]. Lastly, as all of our participants were German and none of them identified as belonging to an ethnic minority, the present results may not be generalizable to non-German or ethnic minority samples.

In contrast to similar trials with this group of patients [10, 28], only one adverse effects was reported as well as two cases of exacerbations of suicidality, all of which were resolved by the carefully planned multilevel safety management implemented in the study. However, as some participants dropped out of the intervention without any notice and did not respond to our intensive outreach efforts, we cannot rule out that their dropout was associated with the occurrence of adverse effects. Moreover, the low rate of adverse effects may be related to the fact that none of our participants reported acute self-harm or suicidal ideation at baseline, which were more frequently reported in previous studies [10, 28].

While the findings of this study are promising, it is important to note that the average treatment duration in the iCBT condition was considerably longer than in the supportive online therapy condition. Participants typically required more than a week to complete a session and the associated homework due to various factors, such as lack of motivation, vacations, academic exams, or time management difficulties. Difficulties in adhering to the prescribed treatment schedule are common in online interventions and have also been reported in previous iCBT trials for BDD (e.g., Enander et al., [23, 25] Rautio et al.,, 2023; Schoenenberg et al., [27]. Although these participants did not receive additional treatment modules or guidance beyond what was originally outlined in the study protocol, they may have had a higher number of interactions with the study therapist, potentially influencing the results beyond the intervention itself. However, these interactions primarily consisted of reminders to complete the next session or homework, occasionally incorporating motivational interviewing techniques, making it unlikely that this significantly impacted the study outcomes.

Furthermore, due to the extended treatment duration in the iCBT condition, the likelihood of spontaneous symptom improvement may have been higher in this group compared to the active control condition. However, body dysmorphic disorder is generally considered a chronic condition with a typically persistent symptom course [4, 8], making spontaneous remission unlikely. Moreover, previous research suggests that individuals with body dysmorphic disorder rarely experience significant symptom reductions without targeted therapeutic intervention [57]. Therefore, while the longer treatment duration in the iCBT condition may have contributed to symptom improvements to some extent, it is unlikely that spontaneous remission alone can account for the observed treatment effects. That said, beyond these potential clinical implications, the considerable difference in treatment duration between groups may have also influenced the statistical modeling. However, its exact impact on the results remains difficult to determine.

Aside from the longer treatment duration in the iCBT condition, the treatment manual remained unchanged, which represents a strength of the study, and distinguishes this study from previous trials that implemented additional modules outside of the original study protocol or expanded the treatment options, such as through longer therapy sessions, home visits, and/or an extended treatment duration as needed [56, 10, 28, 14]. This may have influenced not only treatment outcomes but also attrition rates, which were substantially lower in the aforementioned trials. Indeed, some authors of previous studies suggested that the dropout rates may have been substantially higher had they not made flexible changes to the study protocol to accommodate individual needs such as low mood, family conflict, etc [10].

Some limitations of the present study should be noted. Despite extensive recruitment efforts, this study faced considerable challenges in participant engagement and recruitment efficiency. Firstly, the ratio of recruitment time to potential participants registering their interest in the study was higher compared to other iCBT studies [23, 28, 26]. This may reflect the delusionality of appearance beliefs in BDD patients and their frequent preference for cosmetic treatments [17] as well as the limited awareness of BDD in the general population and among professionals in Germany [58]. Additionally, our study did not collaborate with specialized clinics or hospitals that could refer patients to our trial, in contrast to previous studies with BDD samples [10, 28, 14]. Furthermore, participation was not compensated, and in Germany, psychotherapy is covered by statutory health insurance. Accordingly, there was no financial benefit from participating in the study. This may have also contributed to the high dropout rate in the iCBT condition, which exceeded the expected rate of 30% [53] and was substantially higher than in other iCBT studies for BDD [23, 10, 28, 14], with the exception of another German study that reported a similar dropout rate (55.6%; Schoenenberg et al., [27]. As confirmed in a meta-analysis of smartphone-delivered interventions, offering monetary compensation may be a key factor in reducing attrition in internet intervention studies [59]. Furthermore, the research in this area appears to be marked by a profound heterogeneity in terms of defining completers. In the present study, only participants who had completed all 12 sessions of the intervention and consecutive homework assignments, as well as both baseline and posttreatment measures, were defined as completers. In contrast, previous iCBT studies classified completers as those who had participated in at least half of sessions [60, 27] or who had completed the pre- and post-assessments, irrespective of whether they had fully completed the intervention [23, 24, 28]. For example, Enander et al. [24] reported that only 27% of participants in their randomized sample had actually completed the entire iCBT intervention, and Rautio et al. [28] reported that only 32.6% of adolescent participants and 5.3% of associated caregivers had finished all modules. In addition, the proportion of participants with high delusionality of appearance beliefs in our study, who thus had no or poor insight, was substantially higher than in previous iCBT studies for adults or adolescents with BDD (34.1% vs. 10.0– 17.0% [23, 28, 26]. Thus, it can be assumed that participants in our study were significantly more ambivalent in their decision to begin psychotherapy and less motivated to complete the intervention. It is possible that iCBT for BDD requires that participants have at least moderate insight and motivation to successfully complete such interventions. The same might be true for depressive symptoms, which were significantly more pronounced in our study than in previous iCBT trials for BDD [23, 28]. Lastly, treatment barriers specific to the online context, such as the protection of highly personal data, may have influenced attrition and require further investigation [22, 61].

In order to effectively manage the challenges of participant recruitment and attrition in the iCBT condition, we were forced to stop randomization once the active control group reached the required sample size of 16 participants and assign participants directly to the iCBT condition. This decision significantly constrained our randomization process, thereby limiting the internal validity of our study. For instance, participants who enrolled later may systematically differ from those who were randomized at the outset, introducing a selection bias. Selection bias can lead to distorted results when study groups are no longer comparable, making it difficult to attribute differences between them to the different interventions [62]. To address this issue, we examined potential group differences between the randomized and the non-randomized participants in the iCBT condition across all key demographic and clinical variables, like age, gender, current pharmacologic treatment, delusionality, duration of BDD, comorbidity, BDD symptom severity, BDD cognitions, quality of life and depression at baseline. However, no significant group differences were found (all variables *p* >.05). Moreover, we observed no significant differences between participants in the iCBT and the active control conditions (see also Table 1 in manuscript regarding participant characteristics), suggesting that the groups do not differ systematically and are similarly distributed across relevant factors. Therefore, it is highly likely that the substantial differences observed between the iCBT and the active control group at the post-treatment assessment can be attributed to the respective interventions rather than to confounding variables. Additionally, it is important to note that neither the participants nor the diagnosticians were aware that the randomization process had been interrupted. As a result, we can reasonably assume that neither participant expectations nor diagnostician biases were influenced by this procedural constraint.

To reduce attrition in the long term, gamification principles (e.g., a complex, imaginary setting with possibilities to explore and personalize, rewards, social interaction or competition) could be applied, which may encourage participation in digitally inclined adolescents [63]. Moreover, while previous studies with a young BDD sample included parents or caregivers [10, 28, 14], we chose not to include parents or caregivers in the present study. This decision was made to limit feelings of shame when asking caregivers for permission, as shame is the most frequently reported treatment barrier in BDD [15, 16, 17]. Also, as mentioned earlier, we worked with a slightly older group of participants than in previous studies. However, the inclusion of an affirmative support system might have reduced attrition, particularly among younger participants. To increase completion rates, greater flexibility to deviate from the intervention protocol, especially to address comorbid stressors, might also be beneficial. For example, motivation might be facilitated by activating new modules after a predefined period of time as opposed to after completing a module, or by allowing free choice from a set of modules, as suggested by Schoenenberg et al. [27] and implemented by Rautio et al. [28]. Finally, the length of our treatment protocol may have been overwhelming for our young sample. Although the weekly sessions were timed to match the duration of face-to-face therapy (50 min/week), it may be better to spread the therapy modules over a longer period of time.

## Conclusions

Contributing to the paucity of intervention research in adolescent BDD, this study was the first RCT of iCBT in an adolescent BDD sample. The results suggest that the iCBT intervention was effective in reducing BDD and related symptoms and in improving quality of life. Furthermore, the intervention appears to result in better outcomes compared to supportive online therapy with a clinical psychologist, i.e., a conservative control group similar to low-threshold counseling. Some frequently reported treatment barriers, such as logistic barriers, seem to be reduced. This makes ImaginYouth a promising alternative to face-to-face CBT in areas where access to specialized treatment is limited, adding to the much-needed treatment options. However, the relatively low rate of potential participants registering their interest and the high rate of attrition suggest that iCBT for BDD patients may also involve some treatment barriers that need to be better understood. ImaginYouth may not be appropriate for patients with a greater symptom severity and higher levels of delusionality. Nevertheless, it may be useful as part of a stepped care approach, for mild to moderately affected patients with good insight and high motivation for therapy, thus reserving resources for patients with more severe symptoms and higher delusionality.

## Electronic supplementary material

Below is the link to the electronic supplementary material.

**Additional File 1**: This pdf-file includes the results of the per-protocol analysis not reported in detail in the results section (estimated means, standard errors and pairwise within- and between-group comparisons). **Table S1**: Results of the per-protocol analysis: Changes in outcome measures

## Data Availability

All anonymized study data generated and analyzed during the current study, including metadata and study analysis code, are available upon reasonable request from the first author, and will be made publicly available in a repository after publication of the main outcome data.

## Abbreviations

BDD: Body dysmorphic disorder
CBT: Cognitive behavioral therapy
SSRIs: Selective serotonin reuptake inhibitors
RCT: Randomized controlled trial
iCBT: Internet-based cognitive behavioral therapy
