# Supplementary material for: Efficacy of an internet-based, therapist-guided cognitive behavioral therapy intervention for adolescents and young adults with body dysmorphic disorder: a randomized controlled trial
Source: BMC Psychiatry. 2025 Apr 14;25:374. doi: 10.1186/s12888-025-06797-1 (PMC11995464; doi:10.1186/s12888-025-06797-1)
Supplement: Supplementary file 1 — Additional File 1: This pdf-file includes the results of the per-protocol analysis not reported in detail in the results section (estimated means, standard errors and pairwise within- and between-group comparisons). Table S1: Results of the per-protocol analysis: Changes in outcome measures [file 12888_2025_6797_MOESM1_ESM.docx]

Additional file 1 of Efficacy of an internet-based, therapist-guided cognitive behavioral therapy intervention for adolescents and young adults with body dysmorphic disorder: a randomized controlled trial

| **Additional file 1** | | | | | | | | | | | |
| --- | --- | --- | --- | --- | --- | --- | --- | --- | --- | --- | --- |
| *Results of the complete case analysis: Changes in outcome measures* | | | | | | | | | | | |
|  |  |  |  | Within-group differences | | | | Between-group differences | | | |
|  | Pre-treatment | Post-treatment | Follow-up | Pre-treatment to post | | Pre-treatment to follow-up | | Post-treatment | | Follow-up | |
|  | EMM (*SE*) | EMM (*SE*) | EMM (*SE*) | *M*diff (95% CI) | g (95% CI) | *M*diff  (95% CI) | g (95% CI) | *M*diff (95% CI) | g (95% CI) | *M*diff (95% CI) | g (95% CI) |
| BDD-YBOCS | | | | | | | | | | | |
| iCBT | 28.42  (1.64) | 15.92  (2.33) | - | -12.50  (-16.36; -8.64)*** | 1.56  (0.71; 2.37) | - | - | 9.49 (-3.29; 5.79)** | 1.11 (0.32; 1.88) | - | - |
| Active control | 29.67  (1.47) | 25.40  (2.08) | - | -4.27  (-7.72; -0.81)* | 0.72  (0.16; 1.25) | - | - | - | - | - | - |
| BABS | | | | | | | | | | | |
| iCBT | 14. 75 (1.38) | 11.25 (1.32) | - | -3.50  (-5.69; -1.31)** | 0.72  (0.10; 1.31) | - | - | 2.42 (-1.23; 6.06) | 0.57 (-0.17; 1.31) | - | - |
| Active control | 13.67 (1.24) | 13.67 (1.18) | - | 0.00 (-1.99; 1.99) | 0.00 (-0.48; 0.48) | - | - | - | - | - | - |
| FKS | | | | | | | | | | | |
| iCBT | 38.33 (1.83) | 22.50 (2.59) | 20.58 (2.95) | -15.83 (-20.40; -11.27)*** | 1.65 (0.81; 2.46) | -17.75 (-22.58; -12.92)*** | 1.82 (0.89; 2.72) | 9.00 (1.72; 16.28)* | 0.88 (0.13; 1.63) | 10.42 (2.12; 18.72)* | 0.99  (0.18; 1.77) |
| Active control | 36.86 (1.70) | 31.50 (2.40) | 31.00 (2.73) | -5.36 (-9.59; -1.13)* | 1.01 (0.41; 1.59) | -5.86 (-10.33; -1.38)* | 0.76 (0.18; 1.32) |  |  |  |  |
| FKDK conviction | | | | | | | | | | | |
| iCBT | 42.82 (6.08) | 26.86 (5.93) | 28.38 (6.07) | -15.96 (-25.41; -6.52)** | 1.03 (0.36; 1.66) | -14.44 (-23.75; -5.13)** | 1.09 (0.38; 1.76) | 24.57 (7.56; 41.58)** | 0.94 (0.18; 1.69) | 16.86 (-0.55; 34.26) | 0.62  (-0.16; 1.38) |
| Active control | 51.87 (5.84) | 51.43 (5.70) | 45.24 (5.83) | -0.44 (-9.52; 8.64) | 0.12 (-0.36; 0.60) | -6.63 (-15.58; 2.31) | 0.34 (-0.19; 0.86) |  |  |  |  |
| FKDK frequency | | | | | | | | | | | |
| iCBT | 3.02 (0.20) | 2.26 (0.22) | 2.14 (0.23) | -0.77 (-1.07; -0.47)*** | 1.61 (0.75; 2.44) | -0.89 (-1.22; -0.55)*** | 1.36 (0.58; 2.11) | 0.65 (0.03; 1.27)* | 0.73 (-0.03; 1.48) | 0.64 (-0.00; 1.29) | 0.47  (-0.29; 1.22) |
| Active control | 3.07 (0.18) | 2.90 (0.20) | 2.78 (0.21) | -0.17 (-0.44; 0.11) | 0.43 (-0.09; 0.92) | -0.29 (-0.59; 0.03) | 0.47 (-0.08; 1.00) |  |  |  |  |
| PHQ-9 | | | | | | | | | | | |
| iCBT | 11.33 (1.46) | 7.67 (1.84) | 9.08 (1.79) | -3.67 (0.71; 6.62)* | 0.64 (0.06; 1.20) | -2.25 (-0.44; 4.94) | 0.43 (-0.14; 0.97) | 4.41 (-0.86; 9.68) | 0.66 (-0.08; 1.39) | 2.53 (-2.59; 7.65) | 0.37 (-0.38; 1.12) |
| Active control | 12.00 (1.40) | 12.08 (1.77) | 11.62 (1.72) | 0.08 (-2.92; 2.76) | 0.11 (-0.37; 0.59) | -0.39 (-2.20; 2.97) | 0.09 (-0.42; 0.60) |  |  |  |  |
| KINDL-R | | | | | | | | | | | |
| iCBT | 46.70 (3.18) | 56.60 (3.47) | 57.50 (4.30) | 9.90 (3.66; 16.14)** | -0.83 (-1.47; -0.16) | 10.80 (4.33; 17.27)** | -0.91 (-1.56; -0.22) | -9.06 (-18.66; 0.54) | -0.80 (-1.56; -0.04) | -9.35 (-21.24; 2.55) | -0.30 (-1.05; 0.45) |
| Active control | 45.69 (2.79) | 47.54 (3.04) | 48.15 (3.77) | 1.85 (-3.63; 7.32) | 0.12 (-0.36; 0.60) | 2.46 (-3.21; 8.14) | -0.24 (-0.75; 0.29) |  |  |  |  |
| *Note*. iCBT, internet-based cognitive behavioral psychology; BDD-YBOCS, Yale-Brown Obsessive-Compulsive Scale Modified for Body Dysmorphic Disorder; BABS, Brown Assessment of Beliefs Scale; FKS, Body Dysmorphic Symptoms Inventory; FKDK, Questionnaire of body-dysmorphic cognitions; PHQ-9, Patient Health Questionnaire; KINDL-R, Generic quality of life instrument for children and adolescents – revised; EMM, estimated marginal mean; *SE*, standard error; *M*diff, mean group difference. *p<.05, **p<.01, ***p<.001. | | | | | | | | | | | |
